# The relationship between physical activity, physical health, and mental health among older Chinese adults: A scoping review

**DOI:** 10.3389/fpubh.2022.914548

**Published:** 2023-01-06

**Authors:** Ming Yu Claudia Wong, Kai-ling Ou, Pak Kwong Chung, Kei Yee Katie Chui, Chun-qing Zhang

**Affiliations:** ^1^Department of Sport, Physical Education and Health, Hong Kong Baptist University, Kowloon, Hong Kong SAR, China; ^2^Department of Psychology, Sun Yat-sen University, Guangzhou, China

**Keywords:** physical activity, physical health, mental health, older adults, scoping review

## Abstract

The aging Chinese population is growing fast, and the proportion of the population aged 60 years old is projected to reach 28% by 2040, estimated 402 million. With increased life expectancy, the aging population tends to suffer from health risks and diseases, which create a burden on public health policy. Hence, it is essential to promote healthy and active aging, which includes improving older adults' physical and mental capacities and advocating for the achievement of a healthy life expectancy. Despite the rapidly growing aging population in China, there have been no reviews investigating the effect of physical activity on physical and mental health among older Chinese adults. Therefore, the current study aimed to review studies from the past 15 years that illustrate the effect of physical activity on physical and mental health among Chinese older adults. Based on the Preferred Reporting Items for Systematic Reviews and Meta-Analyses (PRISMA) extension for scoping reviews (PRISMA-ScR), this review addresses the associations between physical activity, physical health and mental health among older Chinese adults. A total of 371 studies were included in the scoping review, which covered the relationships between physical activity, physical health and mental health variables. The scoping review also revealed the impact of various kinds of physical activity affecting older adults' physical health, such as functional fitness, body composition, fall risk and balance, and mental health issues, such as depression, anxiety, cognitive function and quality of life. Moreover, studies have identified innovative forms of physical activity as emerging trends in physical activity interventions for older adults. To conclude, this scoping review captured the common effects between physical activity and overall wellbeing, including physical, mental, and cognitive health. Additionally, diverse forms of physical activity intervention, such as group-based and supervised individual interventions, should be supported, and cross-cultural exercise comparisons should be made in future explorations.

## Introduction

The prevalence of the world's aging population is apparent because of the rise in life expectancy in recent decades (1). Although favorable environments in the current century benefit the health of older adults and provide them with opportunities to achieve a healthy life, this is less than adequate. According to the World Health Organization (WHO) (2), the number of people over the age of 60 years is increasing worldwide, there was 1 billion older population, by 2025 the population will be doubled to 2.1 billion. Almost every country in the world is encountering the rapid growth of its aging population, including China. Based on the statistics provided by the Hong Kong Office of the Government Economist (3), the percentage of Hong Kong residents aged 65 or older increased from 8.2% in 1988 to 19.9% in 2018. It is expected that the pace of aging in Hong Kong will accelerate, and this population segment will reach 31.9% in 2038. In 2015, the WHO released a China country assessment report on aging and health. It summarized that China's aging population was growing fast, and the proportion of the population aged 60 and older, which was 12.4% in 2010, is projected to reach 28% in 2040. However, public health policies in China for addressing older adults' equitable access to care services are still not improving people's overall quality of life (4). Notwithstanding the increased life expectancy, the aged population tends to suffer various health risks and diseases.

Aging is a natural and multidimensional progression of human life that leads to a progressive decline in tissue and organ functions, thus putting older adults at a higher risk of disease and mortality. It causes changes in older adults' physiological, psychological and pathological conditions and their social status (5, 6). Due to reduced physical function and mobility, most older adults are highly committed to a sedentary lifestyle and are the most physically inactive population within a community (7). Research studies have suggested that a longer duration of an inactive or sedentary lifestyle can trigger various physical-related diseases and mental health problems, such as overweight or obesity, diabetes, bone and cardiovascular disorders, lower levels of health-related quality of life (8), depression (9) and loneliness. In particular, a longitudinal study of Hong Kong revealed that the physical and mental health of older Hong Kong adults declined over 10 years, as evidenced by chronic diseases, limited functional fitness, and depression. Furthermore, the study also indicated a reciprocal relationship between physical and mental health, with mental health shown to be the most influential factor in changes in physical health (10).

In 2020, the WHO released the “Decade of Healthy Aging Baseline Report” (2), which emphasized the promotion of healthy aging, including improving older adults' intrinsic capacity (both physical and mental capacities) and advocating achieving a healthy life expectancy by 2023. Given that a recent systematic review and meta-analysis on physical activity and healthy aging highlighted that physical activity could increase healthy aging by 39% (11), it is worth summarizing and indicating the benefits of physical activity on physical and mental health as recommended by the WHO. However, no reviews have investigated the effect of physical activity on physical and mental health among older Chinese adults. A preliminary search was conducted and found that there were limited studies and no reviews related to this field on the increasing Chinese aging adult population before 2006. Moreover, including more reasonable studies to find more potential correlations is warranted. Therefore, the current research focuses on reviewing studies from the past 15 years to illustrate the effect of physical activity on physical and mental health among older Chinese adults. Upon reviewing the studies, this study reveals the most effective physical activity in improving Chinese older adults' physical and mental health, indicates the limitations of the literature and provides suggestions to promote healthy aging among the Chinese population.

## Methods

### Protocol

The scoping review was developed according to the Preferred Reporting Items for Systematic Reviews and Meta-Analyses (PRISMA) extension for scoping reviews (PRISMA-ScR) (12).

This scoping review aimed to address the relationship between physical activity, physical health and mental health among older Chinese adults.

### Eligibility criteria

The scoping review identified studies examining the relationships between physical activity, physical health and mental health. To ensure the comprehensive coverage of the studies' outcomes, relevant studies published from 2006 were identified.

The inclusion criteria for the studies were as follows:

1. Chinese adults aged 55 or older in the community and institutional settings (such as education centers, social services centers and clinical settings) who have participated in physical activities.
2. Chinese adults aged 55 or older who were included in cross-sectional, randomized control trial, case–control model, systematic review, meta-analysis, cohort design, and longitudinal studies with a referenced measuring scale.
3. Relevant studies that were written in Chinese or English.

### Search strategy and information sources

The keywords applied to the search were “Physical Activity OR Exercise,” “Older Adults OR Elderly OR Aging People” and “Hong Kong OR China,” which were first established in English and then translated into corresponding Chinese words for searching in the Chinese database. We limited the research to studies that took place from 1 January 2006 to 4 January 2021. In addition, the reference lists of the highlighted systematic reviews were searched and used as a data source.

The identification of relevant studies was conducted in a step-by-step approach. Only studies that met the eligibility criteria were selected for full-text review and synthesis. If there were duplicate publications, the publication with the most inclusive data was selected for further review and meta-analysis.

First, a completely comprehensive search approach was used, which indicated both published research from bibliographic databases and unpublished studies from gray literature. The following bibliographic databases were searched extensively using the stated review questions, selection methods (inclusion and exclusion criteria), and relevant research with the mentioned keywords: JSTOR, SCOPUS, Cochrane library, Ovid EMBASE, MEDLINE (EBSCOhost), SPORTDiscus, Web of Science, PubMed, PsycINFO (ProQuest), and the Chinese database: China Academic Journals Full-Text Database.

Second, based on the eligibility criteria, titles and abstracts were identified, screened, and selected from a list of retrieved publications, research works, and reports. Potentially relevant studies that did not meet the requirements were excluded. Full-text paper versions of all of the prospectively accepted studies were collected to determine whether the selected articles met the inclusion criteria.

### Study records

Mendeley reference management software was used to store and organize all studies. First, we created a folder for scoping review and a subfolder to import all citations from databases. Second, duplicate records were removed using the “check for duplicates” tool. Third, based on the inclusion and exclusion criteria, the titles and abstracts of all relevant papers were reviewed. Fourth, studies were selected for full-text reviewing and retrieval, and researchers used the tool that highlights and annotates content to determine eligible articles. Fifth, articles deemed ineligible were removed from the review process.

**Figure 1:**
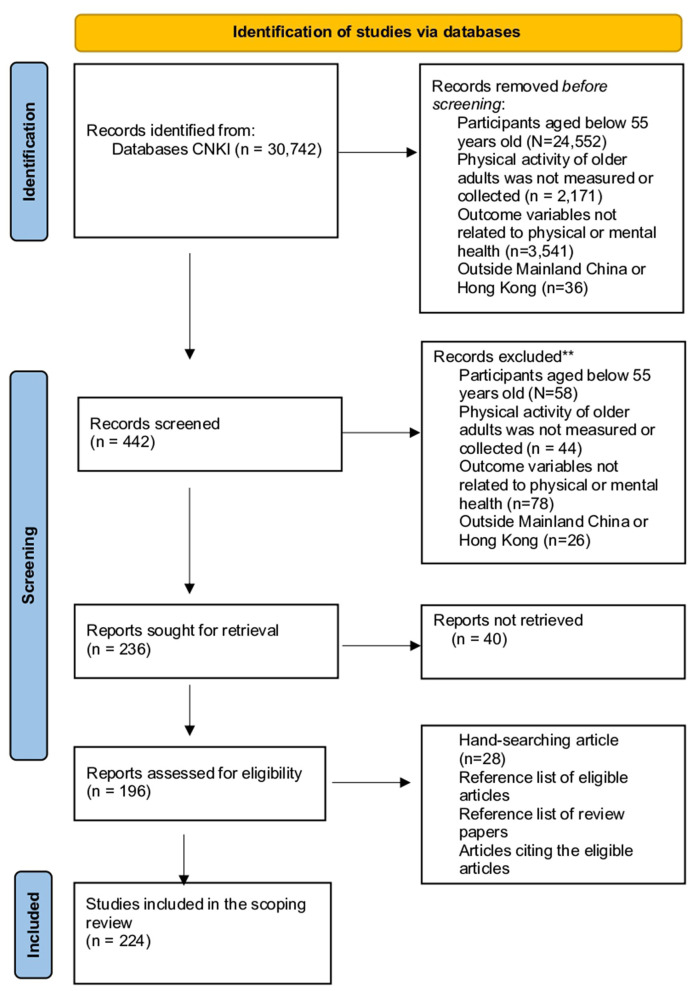
Chinese article screening flowchart.

**Figure 2:**
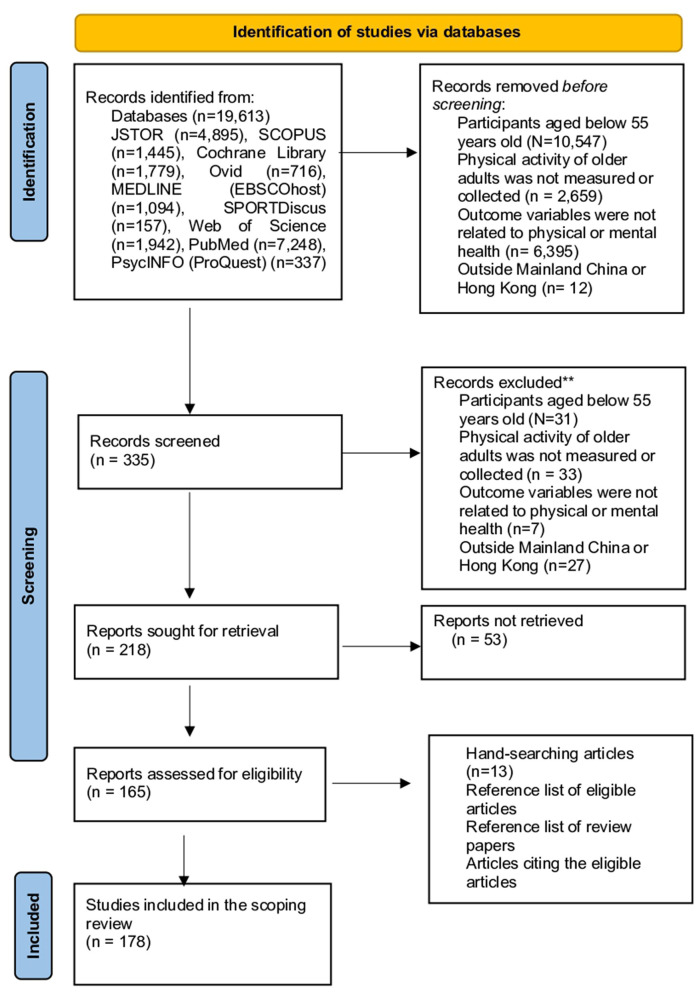
English article screening flowchart.

However, limitations and criticisms arising from the studies, such as sample representativeness, reliability and validity, were further examined using the tools for appropriate risk of bias assessments. Articles that were ineligible were excluded from the review process. A flow diagram of the study identification process is shown in Figures 1, 2.

### Data collection

Three or more academic staff members performed the data collection. The first coder checked and evaluated manuscripts that did not satisfy the eligibility requirements, while the other two coders ensured the correctness of all included articles. Coders resolved controversies through discussion.

The information extracted from the studies included the research design and technique used in each study; the geographical location of the study, including additional demographic information; the type of measurement instrument used to determine the benefit of physical activity; the type of physical exercise that was examined in the research; and statistical findings from physical activity characteristics.

### Outcomes and prioritization

The top priority outcomes were any measure of an older person's physical health or mental wellbeing. Additionally, this review determined that there were fewer priority outcomes that were physical activity features of the evaluated research, such as the common form of physical activity that target populations had engaged in and the type of physical activity intervention, including duration details.

### Synthesis

The synthesis included a descriptive overview and analysis of selected studies' extracted data. In addition, the synthesis included quantitative analysis (i.e., frequency and duration analyses) on the physical activity intervention, the number of physical activities involved, and the measurement employed and qualitative analysis (i.e., content analysis) on the effect of physical activity on different mental health issues, psychological wellbeing, and physical fitness outcomes.

## Results

### Demographic

The 371 studies reviewed included 321 (86.52%) studies from China, 49 (13.21%) studies from Hong Kong and 1 (0.27%) from both China and Hong Kong. Some studies targeted specific types of patients, including 19 on mild cognitive impairment; 12 on knee osteoarthritis; 11 on hypertension; 10 on type 2 diabetes; 6 on heart failure; 5 each on diabetes and depression; 3 each on hypertensive and cardiovascular disease; 2 each on Parkinson's disease, Alzheimer's disease, dementia, anxiety, chronic obstructive pulmonary disease, metabolic syndrome, sarcopenia, low back pain, chronic illness, chronic pain and physical disability; and one each on femoral neck fractures, total hip replacement, cerebral infarction, hyperlipidemia, constipation-predominant irritable bowel syndrome (IBS-C), schizophrenia, acute coronary syndrome, stroke, musculoskeletal pain and nasopharyngeal cancer survivors.

The total population included in these 371 studies was 643,190, including 611,070 (95%) English language studies and 32,120 (5%) Chinese language studies. Out of the total population, 327,109 (50.86%) subjects were female, 278,534 (43.3%) were male, and 37,547 (5.84%) were uncertain or not mentioned.

## Study design

### Intervention study

Among the eligible studies, 225 adopted an intervention study design, 161 were randomized controlled trials, 23 were non-randomized controlled trials, 34 used single-group repeated measures, and 7 were two-group repeated measures. The articles using the intervention design mainly focused on the effect of physical activity on physical health (62.1%), cognitive function (20.9%), quality of life (12.7%), and depression and anxiety (4.35%). The exercises imposed in the studies were Tai Chi (76), Qigong (14), low-impact exercise (8), Baduanjin (18), aerobic exercise (23), dance (12), walking (8), elastic band resistance exercise (6), tennis (5), Otago exercise (4), Diabolo exercise (4), dumbbell exercise (2), light volleyball (3), gait training (1), Chanwuyi (1), Ving Tsun (1), yoga (1), cyber-golfing (1), and aquatic exercise (1).

The physical activity intervention duration varied from study to study. The duration of the Tai Chi intervention studies was ~8 weeks to 1 year, and the duration of the Qigong intervention studies was 8–10 months, with frequencies of ~one to six times per week and for 30–90 min each session. The duration of the low-impact exercise intervention studies was 6–24 weeks, and the intervention frequency was ~two to seven times per week and 25–60 min each session. The duration of the Baduanjin intervention studies was 12–24 weeks, and the intervention frequency was ~three to five times per week for 24–60 min each session. Low-impact exercises and mind–body exercises therefore tended to have a shorter intervention duration. In contrast, the frequency and duration of aerobic exercises were shown to be longer. For example, the duration of the aerobic exercise intervention studies was 8–24 weeks, and the intervention frequency was two to five times per week for 30–90 min per session. The duration of the dance intervention studies was 12–60 weeks, and the intervention frequency was ~two to five times per week for 30–90 min each session. The duration of the walking intervention was 10 weeks to 18 months, with an intervention frequency of two to five times per week and 45–60 min per session. Ball games, including tennis and volleyball, were revealed as significant interventions for improving older adults' health. The intervention duration tended to be 4 weeks to 1 year, with an intervention frequency of three to five times per week and 60–120 min per session. Resistance exercises included the elastic band resistance exercise and the dumbbell exercise interventions. Their study durations were 4–12 weeks, with frequencies of three times per week for 60 min each session. Several special kinds of exercises have also been examined in older adults' exercise intervention studies, including the Otago exercise, gait training, Chanwuyi, Ving Tsun, and cyber-golfing. The durations of these interventions were 4–24 weeks, with a frequency of three times per week for 20–60 min each session. Surprisingly, the yoga intervention study duration was 1 year, and the intervention frequency was three times per week for 60 min each session; there were no short-term yoga intervention studies. Additionally, the duration of the Diabolo exercise was 12 weeks to 12 months, with a frequency of three to five times per week for 45–60 min each session. The aquatic exercise intervention study duration also had a longer duration of 10 weeks and an intervention frequency of twice a week.

### Cross-sectional study

Among the eligible studies, 84 were cross-sectional in design; 26 of the studies investigated physical health, 32 investigated mental health, and 16 investigated cognitive function, while 6 of them investigated both physical health and mental health, 2 investigated both physical health and cognitive function, and 2 of them did not mention either specifically. The articles were mainly focused on examining quality of life (58%), physical health (19.4%), depression (16.1%), and cognitive function (6.5%). The types of exercises utilized in the studies were low-impact exercise (19), Tai Chi (12), aerobic exercise (3), Qigong (2), basketball (1), tennis (1), and square dancing (1); 48 studies did not specify an exercise. Most of the studies used questionnaires [i.e., the International Physical Activity Questionnaire (13), Physical Activity Scale for the Elderly (14), the Chinese version of the International Physical Activity Questionnaire (15), self-reported Activities of Daily Living Scale (16), and the 31-item Yale Physical Activity Scale (17)] to collect the data. Other than the 59.5% of the studies that used questionnaires, 16.45% of the studies used physical fitness tests, such as open exercises, sit-and-stand tests, dumbbell arm curls, chair sit-and-reach tests, back scratch tests, and 8-feet up-and-go tests, 18.7% of the studies used a self-designed survey, and 5.35% of the studies were qualitative research collected during face-to-face interviews.

### Cohort study

Among the eligible studies, 61 articles were cohort studies, and eight articles were longitudinal studies. The cohort study articles mainly focused on physical health (58.35%), cognitive function (29.65%) and quality of life (12%). The articles using longitudinal data were primarily focused on physical health (62.5%), depression (12.5%), quality of life (12.5%), and cognitive function (12.5%). The exercises adopted in the studies were Tai Chi (33), physical exercise (10), square dancing (4), swimming (2), walking (2), outdoor cycling (1), light volleyball (1), and low-impact exercise (2), while 13 of them did not specify the exercise. Most of the studies used the following questionnaires for data collection: the International Physical Activity Questionnaire (13), Beijing Longitudinal Study of Aging Leisure-Time Physical Activity Questionnaire (18), Chinese Health Nutrition Survey (the University of North Carolina, 1989-2011) (19), Physical Activity Scale for the Elderly (14), the Chinese version of the International Physical Activity Questionnaire (15), and Activities of Daily Living Scale (16). Other than studies using questionnaires, 19.8% used a self-designed survey, 33% of the studies used physical fitness tests (e.g., examples of physical fitness tests), and 5.3% of the studies conducted face-to-face interviews.

## Mental health

### Depression

#### The relationships between physical activity and older adults' depression and anxiety

Intervention studies have found that physical activity improves older adults' depression (20–23). The physical activity interventions adopted were aerobic exercise, Tai Chi, walking, low-impact exercise, Otago exercise, Chanwuyi, and Qigong, and these interventions examined the changes they made after a 12–24-week intervention program. The results indicated that Qigong practice can improve depressive symptoms after at least 8 weeks of practice, and individuals' self-esteem can even be enhanced after completing the Qigong intervention (20). Moreover, according to technicians in the mental health rehabilitation field, Chanwuyi was shown to lower the risk of developing dementia, decrease knee pain, improve health-related quality of life, and reduce depression (22). The main research findings were that Otago exercise can help improve depression and anxiety (21). Additionally, a 12-week intervention of Tai Chi practices can effectively improve depression in elderly individuals (23). Few studies have measured physical activities on older adults' anxiety. One study found that older people who engaged in physical activity groups for at least 4 weeks showed improvement in state anxiety, trait anxiety, and anxiety self-assessment scale scores (24). Among the cross-sectional studies, 12 articles mentioned depression. The questionnaires used to measure depression were mainly self-designed standardized questionnaires (25), the Center for Epidemiology Studies-Depression scale (26), the Chinese version of the 36-item Short Form (27), and the self-rating Depression Scale (Geriatric Depression Scale) (28).

Furthermore, among the cross-sectional studies, it was shown that exercising more than three times a week relates to a low prevalence of depression with any type of physical exercise (25). It also demonstrated that regular and long-term practice of Tai Chi boxing can effectively lower the chance of depression for older adults (29) and significantly reduce the severity of depression. The mediation studies also revealed that physical activity, neighborly reciprocity, and sunshine exposure are significant pathways *via* which particulate matter 2.5 (PM2.5) influences depressive symptoms (30). Hence, the above showed that aerobic exercise was linked to fewer depressive symptoms, a better quality of life, and improved cognitive performance (31), and activities of daily living, leisure-time exercise, and physical activity (32), especially in Tai Chi and walking, showed a positive effect in reducing older adults' depression.

### Quality of life

#### The relationship between physical activity and older adults' quality of life

Intervention studies examine the relationship between physical activity and quality of life. The physical activity interventions adopted were 12–26 weeks of aerobic exercise, Tai Chi, dumbbell exercise, Baduanjin, dance, elastic band resistance exercise or the Qigong intervention program. The intervention studies found that older adults showed less perceived stress, a better quality of sleep and a higher confidence level in their self-rated health (33). Overall, their psychological wellbeing improved after participating in physical activity (34). Research has also demonstrated that long-term regular exercise can significantly improve the quality of life of elderly individuals in the community, and choosing a fitness method that suits older adults is an important way for elderly individuals in the community to improve their quality of life (35).

Among the cross-sectional studies, 28 articles mentioned the quality of life. The questionnaires used to measure the quality of life were mainly the Chinese version of the abbreviated Neighborhood Environment Walkability Scales (36), Physical Activity Scale for the Elderly (14), the Chinese version of the Health-Promoting Lifestyle Profile II (37), Chinese Citizen Health Literacy Questionnaire (38), Activities of Daily Living (16), Quality of Wellbeing Scale (39), Chinese Longitudinal Healthy Longevity Surveys (National School of Development of Peking University) (40), the six-item De Jong Gierveld Loneliness Scale (41, 42), Memorial University of Newfoundland Scale of Happiness, Subjective Wellbeing Scale for Old People Participating in Physical Exercise, Michigan Hand Outcomes Questionnaire, self-report questionnaires, World Health Organization Quality of Life (WHOQOL-BREF), and Quality of Life Scale. The results show that physical activity was linked to different kinds of quality of life, such as a reduction in yearly health care costs and improvements in health literacy, older adults' health management and health conditions. Elderly people who took part in physical fitness revealed higher overall scores of happiness (43), and those who participated in physical exercise showed a higher level of quality of life than those who did not (44). Physical exercise was shown to increase the physical pleasure of elderly individuals and provide them with a more comfortable living experience, thus facilitating the improvement of their physical health and subjective wellbeing (45). In addition, older adults who resided in more walkable areas or who were happier with their neighborhood environment spent considerably more time engaging in low-intensity physical activity (46).

Moreover, articles that adopted cohort studies revealed that using low-to-moderate intensity physical activity regularly (i.e., leisure time exercise, Tai Chi, and walking) for a minimum of 3–5 times per week could lead to a decrease in mortality among older adults. Other than low-intensity physical activities, older adults who engaged in moderate physical activity demonstrated a much higher level of quality of life. The older adults' exercise duration, intensity, frequency, and volume had a critical impact on their level of quality of life (47). Furthermore, participants who maintained a high level of physical activity or raised their physical activity from low to high levels had a reduced risk of all-cause death. They were less likely to suffer from cardiovascular disease, so they had a better quality of life (48). People who exercised regularly had a lower risk of dementia and many other conditions, thus increasing their quality of life (49). Regarding the measurement of physical fitness level, recent studies used tests such as flexibility, leg strength, stepping in place with closed eyes, standing-walking, and standing on one foot with closed eyes. The level of quality of life was examined by using questionnaires such as the Quality of Life Questionnaire, self-reports, and 36-Item Short-Form Health Survey.

## Physical health

### The relationship between physical activity and older adults' physical fitness

Intervention studies adopted aerobic exercise, aquatic exercise, Diabolo exercise, Baduanjin, Chanwuyi, elastic band resistance exercise, tennis, cyber-golfing, dancing, gait training, home-based exercise, moderate-intensity exercise, kick shuttlecock, light volleyball, Qigong, strength exercise, Tai Chi, Ving Tsun, walking, and yoga as physical activities to examine. The results from all articles stated that different physical activities could benefit older adults in various ways. The results indicated that Baduanjin has the potential to reduce frailty, increase physical activity, and discourage negative emotions in a safe and effective manner. As a result, Baduanjin is worthy of further popularization and application in frail older individuals as an effective, affordable and safe traditional health care exercise (50). They found that older women who engaged in low-intensity physical exercise showed less bone mineral density (BMD) loss in their hips than those who did not participate (51). Additionally, the Qianlong regimen Dhyana can enhance body symptoms and organ function in older women and their ability to adjust and improve their emotional and mental states (52). Older adults who engaged in regular exercises had significant changes in body composition, with a particular reduction in abdominal fat (53). Diabolo is known as the Chinese yo-yo. There is a thin cylinder connected to the wheel of the Chinese yo-yo Diabolo, and the string can be wrapped on it to shake and rotate the whole Chinese Diabolo (54). Diabolo exercise significantly lowers blood pressure in older people with moderate primary hypertension. Diabolo is also appropriate for chronically ill individuals and those with a weak constitution (55). The 12-week wheelchair Tai Chi ball exercise intervention improved upper extremity muscular strength and is a realistic exercise for older adults with disabilities (56). The 12-week progressive resistance exercise with elastic bands might enhance the elderly's body composition, particularly by increasing lean mass. In addition, a mix of aerobic and resistance training was also shown to be helpful in increasing isokinetic muscular strength in the lower extremity joints (57) and preventing age-related muscle deterioration (58). Eight weeks of squat training enhances the motion sensor of knee flexion in knee osteoarthritis patients. A year of yoga intervention can help lower waist circumference and systolic blood pressure for older adults (59). Air volleyball has a favorable influence on the physical quality of elderly people in terms of strength, flexibility, and agility (60). In community-dwelling older individuals, 12 weeks of low-intensity physical activity (such as Tai Chi or walking) can help reduce body fat, enhance physical fitness, lower fall risks (31), increase BMD and reduce the risk of osteoporosis in elderly individuals (61).

Among cross-sectional studies' outcomes, studies showed that physical activity was linked to physical function improvement. Vigorous physical activity was related to a graded decrease in the risk of stroke compared to low-intensity physical activity. Commuting physical activity, such as riding a bicycle and walking, was linked with a 20–45% reduction in the incidence of various aberrant metabolic syndrome components in women. Multivariable-adjusted logistic regression analysis showed that participants with higher levels of overall physical activity had a reduced chance of having metabolic syndrome (62). Scholards (63) discovered a significantly lower risk of hypertension (a 20–45% reduction) and coronary heart disease (35–55% reduction) among those who participated in moderate to high levels of walking/square dancing or morning exercising/Tai Chi and a lower risk of stroke (25% reduction) among those who participated in moderate to high levels of walking/square dancing. Moreover, vigorous physical activity appeared to be more significant than moderate and mild activity in inducing positive effects on hypertension prevention (64). Studies have shown that the better the physical exercise behavior among hypertensive older adults aged 60 years or older in urban communities is, the greater the effect on their physical fitness; in other words, people with a better physical lifestyle have a larger improvement in their functional fitness status (65). Low-intensity physical activity (such as Tai Chi and walking) can aid in the treatment of cardiovascular disease (66). Moreover, air volleyball is more suitable for older individuals than traditional volleyball, and it has been shown to improve their physical health, including regulating fat buildup in internal organs to some extent and enhancing cardiopulmonary function and physical fitness in both sexes (67).

In cohort studies, there were 17 articles that mentioned physical fitness related to Tai Chi, leisure time exercise, walking, square dancing and swimming. The results revealed that Tai Chi could efficiently reduce body fat and enhance lean body mass among elderly individuals, thus attaining the goal of body strengthening. Tai Chi can also help to maintain the ventilation function of the lungs and the blood supply capacity of the heart, as well as treat the decreased heart and lung function caused by age (68). On the other hand, aerobic exercise can considerably enhance the cardiovascular function of the elderly population, with low-to-moderate-intensity exercise being more suitable and effective for them (69). Swimming, as another aerobic sport, significantly facilitates losing weight, lowering waist circumference, and avoiding the illnesses and discomfort that come with obesity. Elderly individuals' cardiovascular systems and lung function were enhanced, as was their capacity to exercise. More significantly, consistent swimming can successfully treat atherosclerosis. Swimming significantly enhanced the physical condition of the elderly and should be actively promoted in fitness and community activities (70).

### The relationship between physical activity and older adults' physical health problems

Intervention studies that adopted Tai Chi, Qigong, and walking as physical activities produced an evidence-based fall prevention program that could be applied in community settings to enhance physical fitness and minimize fall risks in community-dwelling older persons (71). In older individuals with type 2 diabetes mellitus and adult-onset diabetes, the Kinect-based Kaimai-style Qigong intervention successfully lowered glycated hemoglobin and enhanced balance and cognitive function (72). In individuals with non-specific chronic low back pain, Tai Chi was found to reduce pain but not enhance lower limb proprioception (73). In addition to different kinds of physical activity interventions, the Modified Eighth Section of Eight-Section Brocade is considered an effective exercise-based therapy for reducing symptoms and indices in post-menopausal women. The low attrition and high exercise compliance in the therapeutic intervention showed that Modified Eighth Section exercise is safe, practical, and well-tolerated among post-menopausal women (74).

In the cohort studies, 10 articles mentioned the effect of Tai Chi, leisure time exercise, outdoor cycling exercise, dance and walking in improving physical health. The results showed that under low lighting, Tai Chi subjects enhanced their foot clearance, head inclination angle, and center of pressure displacement to increase their stability during stair climbing (75). As measured by the Leisure-Time Physical Activity Questionnaire score, low physical activity was linked to frailty, impairment, poor physical function, and an increased risk of death (49). In silent standing, the Tai Chi group had a significantly longer postural time to touch and a significantly shorter postural time to contact in fitting activities (75). Other than Tai Chi, sport dancing was found to be effective in increasing lower limb strength and improving the integrated exercise function of the lower limb in male seniors, which is advantageous in delaying muscle aging and reducing falls. The effects of sport dancing on BMD are associated with muscular functions in the elderly population. Sport dancing increases BMD and decreases osteoporosis, which is beneficial to the prevention and treatment of senile osteoporosis (76). The study also found that following a fitness line dancing exercise program can significantly enhance different markers of physical and mental health in middle-aged and older women. It is beneficial to middle-aged and older women's fitness. Furthermore, outdoor cycling is beneficial for elderly individuals' physical self-esteem and mood state (77). In addition to daily exercise engagement, horticultural therapy activities are shown to enhance older adults' enjoyment of life and improve their physicality and mental state, promote the recovery of body function, and improve quality of life, making them more optimistic in the face of old age (78). Exercise can help seniors avoid mental problems, increase muscular grip and cardiovascular and immune function, and contribute to their overall physical and mental health (79).

## Cognitive function

### The relationship between physical activity and older adults' cognitive health

Intervention studies that have examined the relationship between physical activity and cognitive function have examined exercises such as Baduanjin, Qigong, Tai Chi, aerobic exercise, dance, walking, and dumbbell exercise, and the changes among participants were tested after 12 weeks to 1 year of initiation of the intervention program. Among the intervention study outcomes, older adults who continuously engaged in Tai Chi exercise for a year showed a significant reduction in the chance of acquiring dementia (80). Six months of Baduanjin training enhanced global cognitive function and memory in older adults with moderate cognitive impairment (81); they also had substantial gains in balance and functional mobility (82), yet Tai Chi resulted in a much higher increase in cognitive function (31). Hence, both Baduanjin and Tai Chi have been shown to help prevent cognitive deterioration and improve memory function (83). Furthermore, Qigong and Wuqinxi exercise significantly improved elderly individuals' immediate memory, spatial structure, speech function, attention, time-delay memory, and executive control functions, with speech function and attention being the most important (84). Aerobic gymnastics can enhance the cognitive function of elderly individuals with mild dementia and prevent the onset of senile dementia (85). Dancing (86) and square dancing (87) significantly positively influence cognitive function in elderly individuals, and they are worthy of widespread adoption and use.

Among the cross-sectional studies, 16 articles measured cognitive health. The subjects either answered questionnaires [i.e., the Shanghai Cognitive Activities Scale, the Spatial Working Memory Span Test (88), Programming of the Psychtoolbox 3.0 Toolbox based on the MATLAB platform (89), the Montreal Cognitive Assessment (90), the Alzheimer's Disease Assessment Scale–Cognitive Subscale (91), and the Chinese version of the Mini-Mental State Examination (92)] or took a test (Delayed Word Recall Test) (42) and underwent magnetic resonance imaging (93). The results showed that a higher degree of late-life leisure activity involvement, particularly in intellectual pursuits, was found to be substantially related to the improvement of cognitive function in elderly individuals (94). Tai Chi outperformed the other physical activities in episodic memory (95) by significantly aiding cognitive specialization and functional integration (96). The results also indicate that basketball at medium intensity is seen as most effective in increasing the cognitive abilities of elderly individuals, and all genders should engage in different levels of basketball activity (97). Open-skill exercises can help elderly individuals improve their memory because they remove irrelevant visuospatial information rather than the ability to resist natural fading (21). Within Chinese aging cohort studies, physical activity improved cognition, particularly visuospatial ability, and lowered the chance of developing dementia (98).

Cohort study articles that mentioned the relation between physical activity and cognitive health tended to adopt low-intensity physical activities such as Tai Chi, dancing and aerobic exercise. The subjects took tests (99) [i.e., event-related potentials (ERPs), the Flanker paradigm ERP P300 test with 2-back tasks and more-odd shifting tasks, the Verbal Fluency Test for Dementia Screening, the differential diagnosis 20 (DDX-20) computer multifunction psychological and physical ability tester, the BD-2-310 concentration tester, rotation appearance test cards, the dynamic balancing machine test, the gait test, and the static balance test] and answered questionnaires [i.e., the Montreal Cognitive Assessment Scale, self-report questionnaires, the Chinese version of the Rivermead Behavioral Memory Test (100), the Hong Kong List Learning Test (101), the Alzheimer's Disease Assessment Scale–Cognitive Subscale (91), the Category Verbal Fluency Test (102), and the Hong Kong version of the Montreal Cognitive Assessment (103)]. The cohort study results found that older adults who exercised on a regular basis had a significantly decreased risk of acquiring dementia than those who did not exercise on a regular basis (104). The outcomes also revealed that exercise can help to postpone the deterioration of cognitive function caused by aging, particularly in terms of episodic memory and executive function (105). In most subtests, the Tai Chi group outperformed the other exercise groups (106). Long-term Tai Chi practice can help middle-aged and older adults maintain and improve their cognitive abilities (107). Tai Chi, ballroom dancing, and walking activities were found to be beneficial in reducing static balance loss and enhancing static balance in the aging population (55, 108). Additionally, most cognitive assessments were higher in the aerobic and mind–body exercise groups with lengthier exercise habits of more than 5 years, and the beneficial outcomes were particularly pronounced in the 65- to 75-year-old age group (109).

## Others

In addition to the findings demonstrated in the previous sections, there were other findings that were not commonly investigated. The authors considered them worth noting, and they could inspire further investigation.

Studies have stated that recency, frequency, and the multidomain approach of physical activities are both possible and helpful for older people with mild cognitive impairment. Individualized maximal fat oxidation (FATmax) training can also help elderly adults with type 2 diabetes improve their everyday physical ability (53). Among older adults suffering from knee osteoarthritis, a strength exercise program might enhance exercise adherence, self-efficacy, decisional balance, knee osteoarthritis symptoms, and physical functioning (66). However, among individuals who suffer from chronic non-specific low back pain, Tai Chi was found to decrease pain but not to enhance lower limb proprioception (73). Additionally, Tai Chi generated more significant physical and mental benefits from long-term exercise engagement, while its short-term effects were less significant (110). Long-term Tai Chi exercises can help older adults enhance their implicit learning abilities (111), and Tai Chi softball has a positive effect on the treatment of senile dementia (112). Additionally, a study also indicated that cyber-golfing might be a viable alternative to golf in terms of improving balance ability among community-dwelling seniors; therefore, cyber-golfing can also be considered a potential therapeutic intervention for rehabilitation in elderly individuals (110).

For older patients with femoral neck fractures, the Otago exercise intervention program can reduce the fear of falling and decrease their anxiety and depression (21), thus improving the fall performance of elderly patients with cerebral infarction, activities of daily living, and quality of life; thus, it is worthy of being widely implemented in the rehabilitation process of elderly patients with cerebral infarction (113). A 12-week high cognitive demand exercise intervention can considerably enhance elderly individuals' cognitive task performance. Fitness dancing has been shown to successfully decrease cognitive deterioration in the elderly population (84), and square dancing has been shown to increase cognitive performance in older adults (87). Finger exercise training can help elderly individuals with mild cognitive impairment improve their cognitive function (35).

According to findings in cross-sectional and cohort studies, a high risk of type 2 diabetes was shown in urban men in northern China (114). Studies also indicated that physical activity is related to a lower incidence of metabolic syndrome and a healthier profile of inflammatory factors and adipocytokines in Chinese individuals (115). In middle-aged and older Chinese individuals, jogging, Tai Chi, and dance are related to a considerably decreased risk of metabolic syndrome (116). Moreover, long-term regular Tai Chi exercise improved the neuromuscular response of the spinae erector and tibialis anterior to lateral perturbation, which will aid in the prompt correction of lateral postural distributions (117).

Other than the additional physical-related findings, different kinds of physical activity interventions and cross-sectional studies have also indicated additional findings in terms of mental-related outcomes. Long-term practice of various forms of physical activities impacted brain functional networks and brain functional plasticity differently in older women (118). In general, physical activity was able to minimize or postpone deterioration of cognitive function caused by aging, particularly in terms of episodic memory and executive function (105), as well as the development of Alzheimer's disease, and an active lifestyle can help avoid the development of mental health problems (119). For example, participation in late-life intellectual activities was linked to improved cognitive function among Hong Kong's community-dwelling elderly population (94). Regular Qigong practice may alleviate sadness, boost self-efficacy, and enhance personal wellbeing for older people suffering from chronic physical disease and depression (20). Furthermore, scholars have indicated that the improvement in health literacy has the potential to improve health management and health status (49).

Table 1 shows the specific outcomes related to physical, mental and cognitive health that are affected by physical activity.

**Table 1:** The relationship between physical activity and mental and cognitive health-related outcomes.

**Physical health outcome**	**Mental health outcome**	**Cognitive function outcome**
• Cardiovascular disease prevention • Cancer prevention • Enhance body composition • Enhance stability • Enhance strength, flexibility, and agility • Enhance functional fitness status • Enhance cardiopulmonary function • Enhance lean body mass • Enhance lung function • Enhance and strengthen proprioceptive function • Enhance physical stability • Enhance nervous system reaction speed • Enhance respiratory function • Enhance body symptoms • Enhance organ function • Enhance physical function • Hypertension prevention • Increase lean mass • Increase isokinetic muscular strength • Lower body fat • Upper extremity muscular strength • Lower fall risks • Knee flexion • Less bone mineral density loss • Lowered glycated hemoglobin • Lower systolic blood pressure • Lower waist circumference • Maintain the ventilation function of the lungs • Maintain the blood supply capacity of the heart • Moderate drop in blood pressure • Prevent age-related muscle deterioration • Reduce metabolic syndrome • Reduce mortality • Reduce pain • Reducing symptoms and indices in post-menopausal • Reducing heart attack • Reducing stroke • Reduce the risk of osteoporosis • Regulating fat build up in internal organs • Reduce frailty • Treat atherosclerosis	• Better quality of life • Happy • Higher level of confidence • Improve psychological wellbeing • Less stress • Reduce depression • Reduce anxiety • Improve self-esteem	• Improve cognitive performance • Improve memory function • Improve episodic memory • Improve visuospatial ability • Improve immediate memory • Improve speech function • Improve time-delay memory • Improve attention • Improve episodic memory • Improve executive function • Lower the risk of dementia

## Comparison between English and Chinese articles

### Methodology

In the intervention study, there were more Chinese language articles (143) than English articles (84). Within the study design, Chinese articles used more randomized controlled trials and single-group repeated measures than English articles. The most frequently used independent variable (physical activity) in both language articles was Tai Chi. However, there are some sports that only appear in English articles, such as gait training, Chanwuyi, Ving Tsun, yoga, cyber-golfing, and aquatic exercise, and some only in Chinese articles, such as elastic band resistance exercise, Otago exercise, Diabolo exercise, and dumbbell exercise. English articles (55) reported a larger number of cross-sectional studies than Chinese articles (32). In the cohort studies, there were more Chinese articles (52) than English articles (20), but most of the longitudinal studies were in English.

### Mental health

In this scoping review, depression, anxiety, and quality of life were seen to be the main focus of mental health among Chinese older adults. However, it is noteworthy that the English articles only measured depression, while Chinese articles only measured anxiety. Only intervention and cross-sectional studies introduced depression and anxiety. English and Chinese articles had similar results in which they found that continuously performing low-intensity exercise (such as Tai Chi, Qigong, Chanwuyi, and Otago) for a long time can improve or lower the chance of elderly individuals developing depression and anxiety. Studies have also discovered that the environment, neighborhood, and social circle are the factors that influence elderly people's depression and anxiety levels.

### Physical health

Among the reviews on physical fitness and physical health, both types of articles measured how physical activities help the elderly in their cardiovascular health and body shape, as well as how regular physical activities help to change body composition and reduce abdominal fat. While English articles introduced physical health by using intervention and cohort studies, Chinese articles contained all three methods.

### Cognitive function

Both English and Chinese articles showed various kinds of physical activities, such as Tai Chi, Qigong, Baduanjin, and Wuqinxi, as helping to postpone the deterioration of cognitive function caused by aging, particularly in terms of episodic memory and executive function.

## Discussion

This is the first scoping review to summarize the relationship between older adults' physical activity participation, mental health, and physical health over 10 years in Hong Kong and mainland China. Overall, 371 eligible studies were included, and all of these studies reported positive findings, indicating that older adults' participation in physical activity can significantly improve their mental and physical health. The following section will discuss and evaluate the general patterns of findings. Some implications will also be suggested based on the findings. Six main categories, including demographic differences in physical activity, types of physical activity, measures of physical activity, mental health benefits of physical activity, cognitive function benefits of physical activity, and physical health benefits of physical activity, will be discussed.

### Demographic differences in physical activity

The age range of the majority of study participants in these studies was 60 to 79. Moreover, most participants were healthy and active older adults. Only a few studies have investigated the relationship between physical activity and participants with cognitive impairment, mental health disorders, or chronic diseases in China. In addition, an absence of reviewed studies included other special groups, such as older adults living alone. Future studies should examine different biopsychosocial factors across various ethnic groups, urban–rural settings, and residence patterns (120).

### Types of physical activity

Generally, the majority of physical activities in these review studies are low-impact exercises, including Tai Chi, Baduanjin, Qigong, walking, Otago exercise, gait training, Ving Tsun, and yoga. Echoing a previous review in high-income countries, community-dwelling older adults preferred to participate in low-to-moderate intensity activities (121, 122). Specifically, Tai Chi was the most common mind–body exercise identified in China and Hong Kong studies, followed by Qigong to improve older adults' physical and mental health, which is in line with recent reviews (9, 122, 123). Unlike previous reviews, this review only introduced Baduanjin and Ving Tsun, which were uniquely Chinese mind–body exercises applied as interventions for older adults. In the future, more Chinese mind–body exercise practices are suggested to be adopted in Western countries to measure their effectiveness on the physical and mental health of older adults.

Regarding other low-impact exercises, relatively few studies conducted walking programs in this review. Other reviews have indicated that walking is a common physical activity for older adults (122, 123), especially in Western countries (124, 125). Most walking programs in the review studies were clinical interventions for older adults with chronic or cognitive diseases; therefore, future studies could build in more walking programs for healthy older adults. Few studies have examined targeted balance training exercises for older adults aimed at fall prevention, such as gait training, Otago exercises, and aquatic exercise. Echoing WHO guidelines on physical activity and sedentary behaviors (126), further research is required to establish the effectiveness of targeted exercises in balance and gait training to prevent falling for older adults (127, 128).

Although it is in line with previous reviews that less vigorous activities were most common for older adults (121), studies also mentioned aerobic and resistance training. However, in contrast to previous reviews (122, 123), fewer studies in Hong Kong and China have targeted resistance training exercises for older adults. According to the latest international exercise recommendations for older adults, aerobic and resistance training are highly recommended for disease prevention (128). Therefore, future studies are suggested to develop more targeted aerobic and resistance training for Chinese older adults.

One emerging exercise mentioned in this review, light volleyball, a modified traditional volleyball, was positive in older adults (129). Providing innovative approaches to exercises in communities can address the demand for alternative physical activities for older adults (130). Therefore, more research is needed to identify new physical activities for older adults, and more samples should be recruited in future studies to obtain sufficient power and reach generalizable conclusions.

### Measure of physical activity

Regarding physical activity measures, most studies used functional fitness tests, followed by self-report scales and face-to-face interviews, which is in line with previous studies (9, 122, 131). Regarding the outcome measure, no studies in this review used longitudinal measures, and the longest duration among these studies was 1.5 years. This result partly echoes a previous review (122), in which most of the reviewed studies measured primary interventions rather than participants' longitudinal engagement. It has been reported that exploring the long-term effects of interventions can determine more significant outcomes beyond merely investigating physical benefits and strategies (123). In the future, more longitudinal measures are needed. Finally, most of the studies were clinically based measurements or sport-specific and focused on traditional older adults' sports. Community sporting organizations can benefit from understanding the trends and determinants of participation to better tailor specific products and programs to the needs of older adults (132).

### Physical health benefits of physical activity

It is indisputable that exercise training in older adults has been associated with health benefits such as decreased cardiovascular mortality, stroke, diabetes, some types of cancer, and maintaining healthy body composition (133, 134). To facilitate older people's daily amount of exercise, review studies mentioned that a physical-activity-friendly environment or outdoor recreation can motivate older adults to exercise more (135, 136). In addition, engaging in physical activity in an aesthetic climate such as horticultural therapy activities can also increase older adults' recreational physical activity (137). Future studies could consider exploring older adults' perception of community or neighborhood physical activity environments and incorporating considerations such as walkability, accessibility, safety, and aesthetics in local urban areas rather than encouraging single-exercise interventions (138).

Exercises programs with theoretical and specialist support, such as research-based and professional instructors, effectively improve health symptoms in different groups. Therefore, it is vital to promote healthy and dignified aging by assisting health care systems in efficiently implementing evidence-based exercise programs for older adults across levels of frailty in community and institutional settings (139). Moreover, further intervention could include supervised physical activity intervention by recruiting specialist supervisors to monitor older adults' safety (e.g., fall risk) (123, 140).

### Mental health benefits of physical activity

Depression and anxiety are the two main disorders investigated in this scoping review. Generally, the lower proportion of studies in this review analyzed the relationship between physical activity and mental health among older adults compared with functional fitness. More surveys are required to analyze older adults' physical activity and psychological outcomes. Furthermore, all types of physical activity examined in the review found significant benefits for older adults' mental health. In particular, low-to-moderate intensity exercises, such as Tai Chi, walking, daily leisure exercise, and Otago, were observed to have positive effects on older Chinese adults' mental health, which is supported by a previous analysis (141).

Notably, one study evaluated the relationship between neighborhood walkability and older adults' mental health (46) and found that environment, neighborhood, and social circles are the effective factors that increased older adults' motivation to engage in physical activity and decreased depression and anxiety levels. This finding echoes a previous study that showed that older adults spend more time in their local areas than other age groups (142). These discoveries suggest that more research should be conducted to investigate the physical activity environment (e.g., walkability) and develop more community-based physical activity programs for older adults. Moreover, it was reported that integrating physical activity into nature, for example, nature-based physical activity, can be more accessible, meaningful, and sustainable for older adults and people with mental illnesses (143, 144). Future studies can consider integrating physical activity into nature for older adults.

Health literacy is another major factor related to older adults' quality of life. The reviewed studies reported that participating in physical activity can increase older adults' health literacy, thus enhancing their health management. This is because inadequate health knowledge among older adults may affect their self-efficacy to make deliberate health behavior choices (145). Older adults with adequate health literacy engage in more physical activity weekly than those with inadequate health literacy (146). In the future, more sophisticated theory-based interventions, for example, those that combine health or physical literacy with physical activity interventions, should be planned for older adults across levels of frailty with different ethnicities, cultures, and languages (147, 148).

### Cognitive function benefits of physical activity

The reviewed articles revealed that low-intensity physical activity exercises, such as mind–body exercises, offer great potential in preventing older adults' memory loss, visuospatial ability, speech function, attention, and control functions, thus enhancing their cognitive function. Traditional physical activity can improve older adults' cognitive functions, as confirmed by previous reviews in China (120, 149). In addition, Tai Chi, Baduanjin, and Qigong, traditional Chinese mind–body exercises, were most frequently included in this review. Dhyana, which combines meditative states with body movement to regulate mental focus and control breathing (150), is also a safe and effective exercise for enhancing cognitive function among older adults (95). However, because most of the participants in the review articles were healthy, more samples are needed to examine how physical activity works as a mechanism to improve cognitive function for older Chinese adults with cognitive-related impairment. For example, combined cognitive and physical exercise training effectively improves objective cognitive function in older adults with subjective cognitive decline in the earliest prodromal stage of Alzheimer's disease (151). It is worth mentioning that few studies have developed multiple-modality exercises with mind–motor training, such as high cognitive demand exercise intervention, Tai Chi softball, and cyber-golfing. Future studies can develop more cognitive training-based physical activity programs using dual tasks, visual memory, or technology to monitor their progress.

## Conclusion

To conclude, the scoping review was able to capture the common effects between physical activity and overall wellbeing, including physical, mental, and cognitive health, and suggested future research endeavors. For example, increasing physical-related research and interventions aimed at the clinical treatment of older adults, conducting a cross-cultural comparison about the effect of Chinese mind–body exercise on the Chinese and Western populations, and extending intervention designs to balancing and gait training for older adults (going beyond aerobic or anaerobic exercises). Moreover, diverse forms of physical activity interventions and experiments were found in the scoping review, such as sitting light volleyball, aquatics, Tai Chi softball, and other innovative approaches for older adult exercises. Extending the impact and effects of these exercises could have a considerable influence and contribute to the field of physical activity and health. Furthermore, the forms of intervention should be investigated, including the effect of the group-based intervention, supervised intervention, exercise, cognitive training integrated intervention, and theoretical-based intervention, while the forms and patterns of intervention should also be in line with the characteristics and the nature of the older adults.

## Author contributions

MW: conceptualization, study design, and writing—original draft preparation. K-lO: data analysis and writing—original draft preparation and editing. PC: conceptualization, study design, and supervision. KC: data qualitative assessment and writing—original draft preparation. C-qZ: supervision. All authors read and approved the final manuscript.
