# Supplementary material for: The relationship between physical activity, physical health, and mental health among older Chinese adults: A scoping review
Source: Front Public Health. 2023 Jan 6;10:914548. doi: 10.3389/fpubh.2022.914548 (PMC9853435; doi:10.3389/fpubh.2022.914548)
Supplement: Supplementary file 1 [file Table_1.docx]

Table 1. The relationship between physical activity and mental and cognitive health-related outcomes

| Physical health outcome | Mental health outcome | Cognitive function outcome |
| --- | --- | --- |
| - Cardiovascular disease prevention - Cancer prevention - Enhance body composition - Enhance stability - Enhance strength, flexibility, and agility - Enhance functional fitness status - Enhance cardiopulmonary function - Enhance lean body mass - Enhance lung function - Enhance and strengthen proprioceptive function - Enhance physical stability - Enhance nervous system reaction speed - Enhance respiratory function - Enhance body symptoms - Enhance organ function - Enhance physical function - Hypertension prevention - Increase lean mass - Increase isokinetic muscular strength - Lower body fat - Upper extremity muscular strength - Lower fall risks - Knee flexion - Less bone mineral density loss - Lowered glycated hemoglobin - Lower systolic blood pressure - Lower waist circumference - Maintain the ventilation function of the lungs - Maintain the blood supply capacity of the heart - Moderate drop in blood pressure - Prevent age-related muscle deterioration - Reduce metabolic syndrome - Reduce mortality - Reduce pain - Reducing symptoms and indices in postmenopausal - Reducing heart attack - Reducing stroke - Reduce the risk of osteoporosis - Regulating fat build up in internal organs - Reduce frailty - Treat atherosclerosis | - Better quality of life - Happy - Higher level of confidence - Improve psychological well-being - Less stress - Reduce depression - Reduce anxiety - Improve self-esteem | - Improve cognitive performance - Improve memory function - Improve episodic memory - Improve visuospatial ability - Improve immediate memory - Improve speech function - Improve time-delay memory - Improve attention - Improve episodic memory - Improve executive function - Lower the risk of dementia |
